# The MacBlue Binary Transgene (csf1r-gal4VP16/UAS-ECFP) Provides a Novel Marker for Visualisation of Subsets of Monocytes, Macrophages and Dendritic Cells and Responsiveness to CSF1 Administration

**DOI:** 10.1371/journal.pone.0105429

**Published:** 2014-08-19

**Authors:** Kristin A. Sauter, Clare Pridans, Anuj Sehgal, Calum C. Bain, Charlotte Scott, Lindsey Moffat, Rocío Rojo, Ben M. Stutchfield, Claire L. Davies, David S. Donaldson, Kathleen Renault, Barry W. McColl, Alan M. Mowat, Alan Serrels, Margaret C. Frame, Neil A. Mabbott, David A. Hume

**Affiliations:** 1 The Roslin Institute and Royal (Dick) School of Veterinary Studies, University of Edinburgh, Easter Bush, Midlothian, Scotland, United Kingdom; 2 Centre for Immunobiology, Institute of Infection, Immunity and Inflammation, University of Glasgow, Glasgow, Scotland, United Kingdom; 3 MRC Centre for Inflammation Research, The Queen's Medical Research Institute, University of Edinburgh, Edinburgh, Midlothian, Scotland, United Kingdom; 4 Edinburgh Cancer Research Centre, University of Edinburgh, Edinburgh, Midlothian, Scotland, United Kingdom; Istituto Superiore di Sanità, Italy

## Abstract

The MacBlue transgenic mouse uses the *Csf1r* promoter and first intron to drive expression of gal4-VP16, which in turn drives a cointegrated gal4-responsive UAS-ECFP cassette. The *Csf1r* promoter region used contains a deletion of a 150 bp conserved region covering trophoblast and osteoclast-specific transcription start sites. In this study, we examined expression of the transgene in embryos and adult mice. In embryos, ECFP was expressed in the large majority of macrophages derived from the yolk sac, and as the liver became a major site of monocytopoiesis. In adults, ECFP was detected at high levels in both Ly6C^+^ and Ly6C^-^ monocytes and distinguished them from Ly6C^+^, F4/80^+^, CSF1R^+^ immature myeloid cells in peripheral blood. ECFP was also detected in the large majority of microglia and Langerhans cells. However, expression was lost from the majority of tissue macrophages, including Kupffer cells in the liver and F4/80^+^ macrophages of the lung, kidney, spleen and intestine. The small numbers of positive cells isolated from the liver resembled blood monocytes. In the gut, ECFP^+^ cells were identified primarily as classical dendritic cells or blood monocytes in disaggregated cell preparations. Immunohistochemistry showed large numbers of ECFP^+^ cells in the Peyer's patch and isolated lymphoid follicles. The MacBlue transgene was used to investigate the effect of treatment with CSF1-Fc, a form of the growth factor with longer half-life and efficacy. CSF1-Fc massively expanded both the immature myeloid cell (ECFP^−^) and Ly6C^+^ monocyte populations, but had a smaller effect on Ly6C^−^ monocytes. There were proportional increases in ECFP^+^ cells detected in lung and liver, consistent with monocyte infiltration, but no generation of ECFP^+^ Kupffer cells. In the gut, there was selective infiltration of large numbers of cells into the lamina propria and Peyer's patches. We discuss the use of the MacBlue transgene as a marker of monocyte/macrophage/dendritic cell differentiation.

## Introduction

The mononuclear phagocyte system is a family of cells comprising progenitors in the bone marrow (BM), circulating monocytes and tissue macrophages [1], [2]. The proliferation, differentiation and survival of many of these cells depends upon macrophage colony-stimulating factor (CSF1) which mediates its effects through the protein tyrosine kinase receptor, CSF1R [3]–[5]. Although *Csf1r* mRNA is expressed in all myeloid cells, the protein product is present at high levels only in mononuclear phagocyte system (MPS) lineage cells [6]. A *Csf1r*-EGFP (MacGreen) transgene described previously perfectly recapitulates the location of *Csf1r* mRNA and provides a marker for MPS cells in tissues [7]. Recent studies of inbred mice have questioned the role of monocytes as immediate precursors for the maintenance of tissue macrophage numbers [8]–[10] but regardless of their origin, the effects of a blocking antibody against the CSF1R supports the concept that macrophage survival/replacement in most tissues requires continuous CSF1R signalling [11].

CSF1-dependent macrophages have been ascribed many roles in tissue repair and homeostasis [4], [5]. Recombinant CSF1 has been tested in clinical trials for several indications [4], but has not yet found a clinical application. CSF1 has a very short half-life in the circulation of mice (1.6 hours), being cleared from the circulation by CSF1R mediated internalization and degradation by Kupffer cells of the liver [12]. Renal excretion becomes the major mechanism of clearance when the receptor-mediated clearance is saturated. The 150 amino acid active CSF1 protein produced in bacteria is well below the renal clearance threshold of around 68 kDa (the size of albumin), and consequently the majority of any injected bolus dose is rapidly cleared by the kidney. Recent studies have reinvigorated interest in CSF1 as a therapeutic agent in tissue repair [13]–[17]. To enable reinvestigation of therapeutic applications of CSF1, we have increased the half-life by producing a conjugate of pig CSF1 (which is active in mice; [18]) with the Fc region of immunoglobulin [19].

The 7.2 kb *Csf1r* region from the MacGreen transgene contains 3.5 kb of *Csf1r* promoter, the first intron including the critical FIRE element [20] and part of the second exon linked to a EGFP reporter [7]. In peripheral blood [21], and amongst inflammatory populations [22], EGFP^hi^ and EGFP^lo^ myeloid cell populations can be distinguished. The MacGreen transgenic construct also expresses in trophoblasts, which utilise transcription start sites (TSS) within a 150 bp conserved region upstream of the major macrophage TSS cluster. To eliminate trophoblast expression, we removed the 150 bp conserved region. This deletion abolished expression in both EGFP^lo^ myeloid cell populations and in osteoclasts (OCL). The latter cells were found to utilise additional TSS within the deleted region [23]. We used the MacGreen transgene, with the 150 bp internal deletion, to create a binary transgene, in which the *Csf1r* promoter driving the transcription factor, gal4-VP16, is cointegrated with a Gal4-responsive UAS-CFP cassette to produce the MacBlue transgenic line [24]. This approach was derived from genetic studies in drosophila; the principle being that the “driver” line (in this case expressing in macrophages) can be crossed to a target line, in which a UAS sequence is placed upstream of a gene of interest. Crossing this line to a UAS-Schlafen4 transgenic line produced expression of Schlafen4 in macrophages that also expressed ECFP, and produced a myeloproliferative disorder [25]. The MacBlue transgene expression was detected in embryonic macrophages, in blood monocytes, microglia and Langerhans cells, interdigitating cells (classical DC) in spleen and in BM-derived macrophages [25]. In a recent study, the MacBlue transgene was used to monitor the trafficking of blood monocytes from the BM [26]. The line was crossed to a CX3CR1^gfp/+^ transgene to study the effect of CX3CR1 in monocyte mobilisation. In CX3CR1^gfp/+^ mice, the EGFP was detected in large stellate macrophages in the BM, whereas ECFP in the MacBlue mice was more restricted to monocyte-like rounded cells [26]. This study provided the first indication that the ECFP in the MacBlue line was not universally expressed by tissue macrophages. Here we report the surprising finding that the majority of adult tissue macrophages do not express detectable ECFP in the MacBlue transgenic mice, but expression is retained on monocytes and classical DC. The MacBlue transgene thus provides a novel marker for study of monocyte and DC trafficking and mononuclear phagocyte development in tissues.

## Results

### The distribution of ECFP-positive cells in the tissue of the MacBlue mouse

**Figure 1:**
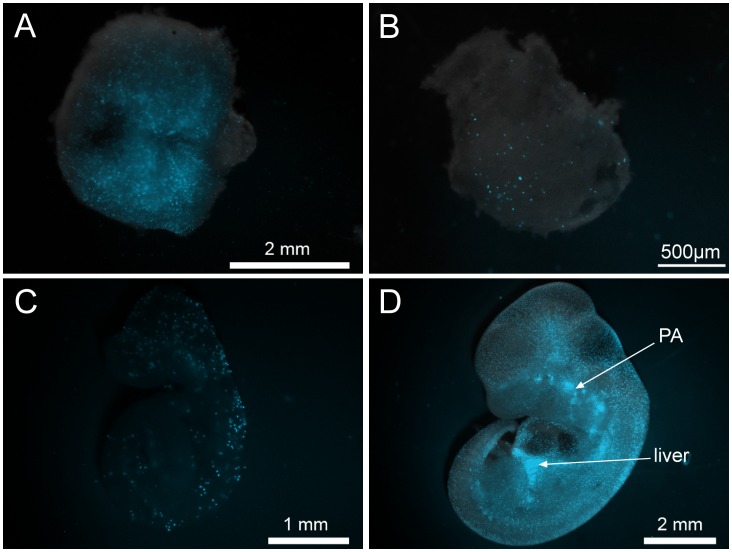
Expression of the MacBlue transgene in the mouse embryo. Embryos and the surrounding membranes were dissected and viewed by fluorescent microscopy. (A) E8.5 decidua from a ECFP^+^ female. The following ECFP expression was paternally derived: (B) E8.5 Reichert's membrane, (C) E9.5 embryo and (D) E11.5 embryo. PA: pharyngeal arches.

The 150 bp deletion of a distal promoter region of *Csf1r* used in the MacBlue ECFP transgene (and the equivalent deletion in the MacGreen) ablates expression in granulocytes, as well as trophoblasts and osteoclasts [23]–[25]. During embryonic development, ECFP^+^ cells in the MacBlue line first appeared in the yolk sac, and subsequently throughout the embryo [24], [25]. We have examined the appearance of the ECFP^+^ cells during embryogenesis in greater detail (Figure 1). ECFP was detectable at high levels in the abundant decidual macrophages (Fig 1A). In view of evidence of an early wave of maternal-derived macrophages during fetal development [27], we examined embryos in which the MacBlue transgene was paternally-derived. The first detectable ECFP^+^ cells were observed in Reichert's membrane (Fig 1B) with occasional cells visible in the yolk sac around 8dpc. Thereafter, the ECFP marker provided a striking view of the progressive infiltration of the embryo by macrophages, including the onset of haemopoiesis in the liver and areas of apoptosis surrounding the pharyngeal arches (Figs 1C and 1D).

**Figure 2:**
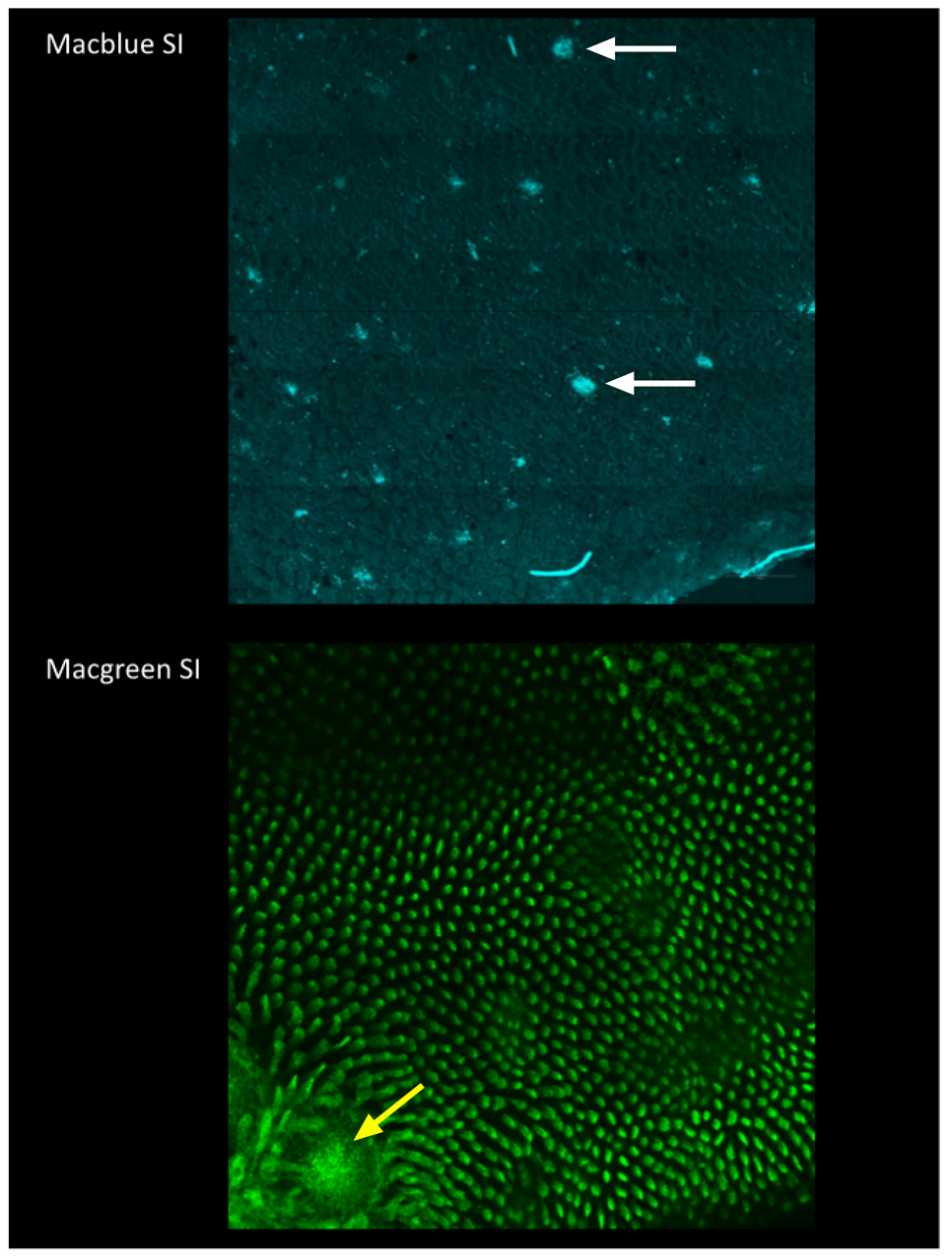
The expression of MacBlue and MacGreen transgenes in the small intestine. En face view of the small intestine of a MacBlue and a MacGreen mouse. White arrows indicate isolated lymphoid follicles (ILF) in the MacBlue, yellow arrow indicates dome region of a Peyer's patch in the MacGreen. The large numbers of EGFP^+^ macrophages in the lamina propria highlight the individual villi in the lower image.

Expression of the MacBlue transgene was previously detected in BM-derived macrophages grown in CSF1, and at high levels in whole mounts of spleen and in Langerhans cells, which can be imaged directly in the ear skin using confocal microscopy [24]. None of the previous studies reported expression in adult organs other than spleen. Lamina propria macrophages are probably the largest pool of macrophages in the body, and in adults are replenished continuously from a Ly6C^+^ monocyte progenitor [28], [29]. They are rapidly and completely depleted by anti-CSF1R treatment [21]. To our surprise, examination of sections indicated that the lamina propria myeloid populations were almost completely devoid of ECFP expression in the MacBlue mouse. Figure 2 shows *en face* views of the small intestinal walls of the MacGreen and MacBlue mice. Whereas the MacGreen transgene highlights the high concentration of CSF1R-dependent macrophages in the villi in the small intestine, and also the concentration of phagocytic cells underlying the dome epithelium of the Peyer's patch, the MacBlue transgene was detectable only in occasional cells in each villus. Interestingly, ECFP^+^ cells were concentrated beneath the epithelia of Peyer's patches and also in isolated lymphoid follicles, and enabled visualization of these structures in whole mounts. The expression of ECFP in the lymphoid follicles is consistent with its expression by interdigitating cells of the spleen [24].

As noted above, ECFP is expressed by Langerhans cells. These cells are also associated with stratified squamous epithelium of the oral and nasal cavity and oesophagus [30]. The ECFP marker also provided a striking marker for a population of stellate cells within the submucosa and muscularis in this site and highlighted the nasal-associated lymphoid tissue (Figure S1).

**Figure 3:**
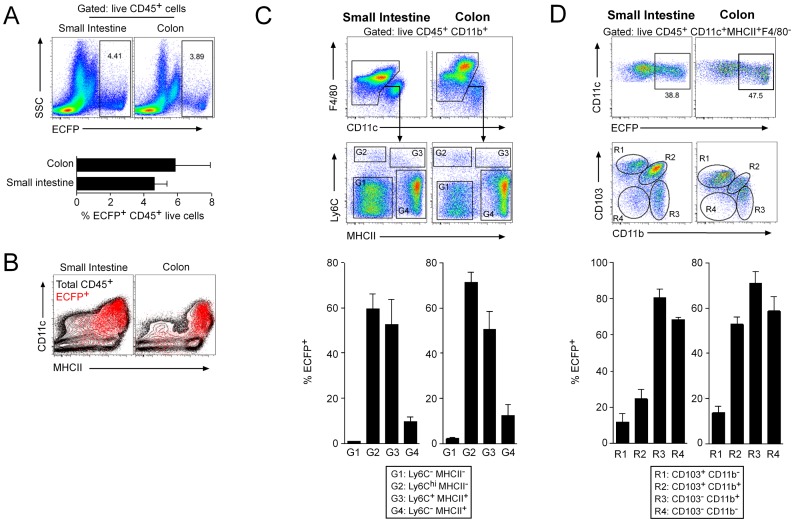
The expression of MacBlue transgenes in the small intestine and colon. Small intestinal and colonic isolates from MacBlue mice were obtained by enzymatic digest and analysed using multi-parameter flow cytometry. (A) ECFP expression in the small intestine and colon (gated on live CD45^+^ cells). (B) Representative FACS plots showing CD45, ECFP, CD11c and MHCII expression in the small intestine and colon of MacBlue mice. (C) Live CD45^+^CD11b^+^F4/80^+^CD11c^−^ cells were split into 4 gates based on Ly6C and MHCII expression. The percentage of ECFP^+^ cells within these 4 gates was examined. (D) The upper FACS plots display representative percentages of ECFP^+^CD11c^+^ cells in the small intestine and colonic isolates. Live CD45^+^CD11c^+^MHCII^+^F4/80^−^ cells were split into 4 regions based on CD103 and CD11b expression. The percentage of ECFP^+^ cells within these 4 regions was examined.

To determine exactly which cells expressed ECFP, we next examined small intestinal and colonic isolates obtained by enzymatic digest using multi-parameter flow cytometry (Figure 3). Consistent with the microscopy, ECFP^+^ cells were relatively rare in the steady state mucosa, comprising 4.6±0.7% and 5.8±2.06% of total live CD45^+^ leukocytes in the small intestine and colon respectively (Fig 3A). The vast majority of ECFP^+^ cells expressed both CD11c and MHCII in both parts of the intestine (Fig 3B). Given that both conventional migratory DC and tissue resident intestinal macrophages in the intestine express CD11c and MHCII, we next used expression of CD45, CD11b, F4/80, CD11c, Ly6C and MHCII to identify DC and monocyte/macrophage subsets to assess their expression of ECFP. Amongst the CD45^+^ CD11b^+^ F4/80^+^ compartment, four populations of cells could be identified on the basis of Ly6C and MHCII expression. F4/80^+^ CD11b^+^ cells lacking expression of Ly6C and MHCII (G1 cells) are eosinophils which display high SSC characteristics [31] and as expected, these were uniformly negative for ECFP expression (Fig 3C). Ly6C^hi^MHCII^−^, Ly6C^+^MHCII^+^ and Ly6C^−^MHCII^+^ myeloid cells (denoted G2, G3 and G4 respectively) represent a monocyte to macrophage differentiation continuum in the intestinal mucosa [28], [29]. The majority of the G2 cells (Ly6C^hi^MHCII^−^ monocytes) expressed ECFP (60% in the small intestine, and 70% in colon, Fig 3C), in keeping with analysis of blood Ly6C^hi^ monocytes (see below). Similarly most Ly6C^+^MHCII^+^ (G3) cells (52.8±10.9%) expressed ECFP at high levels, whereas few (∼10%) of F4/80^hi^MHCII^+^Ly6C^−^ (G4) mature macrophages retain ECFP.

We next examined the expression of ECFP amongst the CD45^+^ CD11c^+^ MHCII^+^ F4/80^−^ population considered classical DC [32]. Around ∼40% of this entire population expressed ECFP (Fig 3D). The intestinal DC pool can be segregated into four discrete populations based on the expression of CD103 and CD11b, all of which have been reported to express the DC-associated transcription factor *Zbtb46* [33]. The majority of ECFP expression is attributable to the CD103^−^ DC subsets, which contained 60–80% ECFP^+^ cells amongst the CD103^−^CD11b^+^ and CD103^−^CD11b^−^ DC subsets in both tissues. The CD103^+^CD11b^+^ DC subset also showed significant ECFP expression with ∼25% of cells positive in small intestine and 55% in colon, whereas fewer CD103^+^CD11b^−^ DC expressed ECFP. All populations also showed a gradient of expression, rather than a sharp peak, suggesting a progressive loss (Figure S2).

Given this pattern of expression, we looked at the corresponding DC subsets in the draining mesenteric lymph node (MLN), using the expression levels of MHCII to define LN-resident (MHCII^+^) and migratory (MHCII^hi^) DC. The expression of ECFP was much lower in the MLN compared with the mucosa. Whereas approximately one quarter of LN-resident CD11b^+^ DC expressed ECFP, of the migratory DC only ∼10% of CD103^+^ CD11b^+^ and CD103^−^CD11b^+^ DC had detectable ECFP, and then only at very low levels compared to the cells in the lamina propria; CD103^+^ CD11b^−^ DC were completely devoid of any ECFP expression (Fig S2).

**Figure 4:**
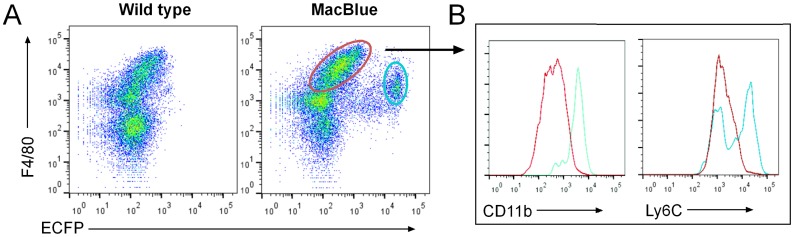
The expression of MacBlue transgenes in the liver. Following digestion of the liver and removal of hepatocytes, cells were examined for ECFP and F4/80 expression in wild type and MacBlue mice (A). F4/80^+^ECFP^−^ (red gate) and F4/80^+^ECFP^+^ (blue gate) populations were then examined for CD11b and Ly6C expression (B).

Consistent with our findings in the gut, there were very few ECFP^+^ cells in liver, kidney and lung compared to the abundant macrophage populations identified with the MacGreen transgene [7] (see also image database on www.macrophages.com). We disaggregated the liver and examined cells of the monocyte/macrophage lineage for the expression of ECFP (Figure 4). The F4/80^hi^ CD11b^lo^ Kupffer cells, which are uniformly EGFP^+^ in the MacGreen mice [7], were devoid of ECFP expression. All ECFP^+^ cells expressed lower levels of F4/80 and high levels of CD11b and had similar distributions of Ly6C^+^ and Ly6C^−^ subpopulations to blood monocytes. Accordingly, we consider that these are blood-derived cells, either migrating in the parenchyma, or trapped in the vessels despite the exsanguination.

**Figure 5:**
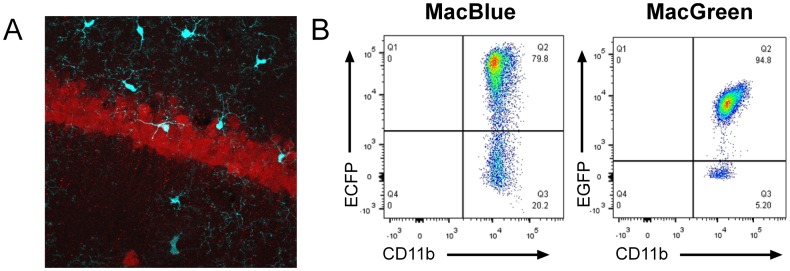
The expression of MacBlue and MacGreen transgenes in the brain. PFA-fixed cryosections from MacBlue mice were immunostained to detect a neuronal marker, neuN (red) in the hippocampus of the brain (A). Microglia demonstrate the ECFP reporter transgene. A representative ×400 z stack projection is shown. (B) Whole brains were digested and analysed for CD11b and transgene expression. Cells were gated on CD11b^+^CD45^lo^.

The MacGreen reporter is expressed in microglia, but is not detected in neuronal cells [7]. Like Langerhans cells, microglia have been reported to be dependent in part upon the alternative CSF1R ligand, interleukin 34 [34]. A recent report suggested that CSF1R can be expressed by neurons, is induced in response to tissue damage, and can mediate trophic effects of CSF1 via direct effects on neurons [16]. The MacBlue ECFP marker was found to be a very effective marker of microglia because the very bright fluorescence and negligible autofluorescence highlights their fine processes. Figure 5A shows a section of the pyramidal layer of the hippocampus of the MacBlue mouse, co-stained for the neuronal marker, NeuN. Occasional ECFP^+^ cells were seen within the pyramidal layer, but they were not NeuN-positive, and had the distinguishing ramified processes characteristic of microglia. To determine whether ECFP was expressed by all microglia, we performed a comparative analysis on disaggregated whole brain from the MacBlue and MacGreen mice. All CD11b^+^CD45^lo^ cells also expressed F4/80 (data not shown). At least 95% of microglia were detected with the EGFP transgene. With ECFP, the distribution was less clearcut, and varied between preparations from 60–80%. The analysis is complicated by a higher level of autofluorescence in the ECFP channel in microglia. The fluorescence showed a rather broad distribution, suggesting that it could be progressively lost (Fig 5B).

### The effect of CSF1 treatment on ECFP^+^ monocyte and macrophage numbers

**Figure 6:**
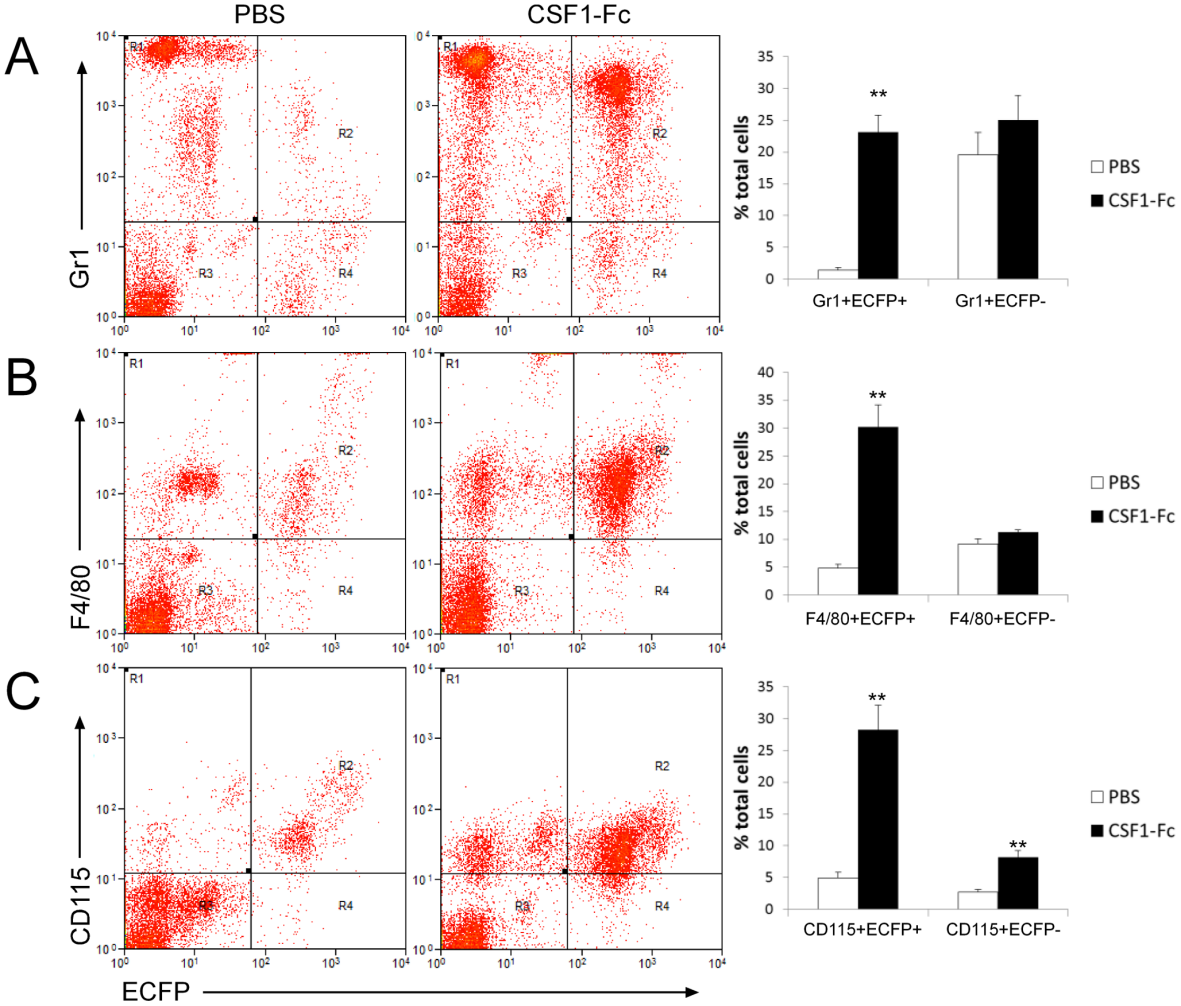
The expression of MacBlue transgenes in the blood of mice injected with CSF1-Fc. Male and female MacBlue 8–15 weeks old were injected subcutaneously with either PBS or 1mg/Kg porcine CSF1-Fc for 4 days prior to sacrifice on day 5. Blood was analysed via FACS for ECFP expression in conjunction with (A) Gr1, (B) F4/80 and (C) CD115 (CSF1R). Representative FACS plots are shown.

Blood monocyte numbers are massively increased in response to administration of CSF1 [35]. CSF1 also controls the maturation of blood monocytes [10], [11] and their infiltration into tissues and the proliferation of tissue macrophages [36]. Bone marrow-derived macrophages grown in CSF1 also expressed the ECFP transgene [24]. We have recently described a novel CSF1-Fc conjugate that is more active than the native molecule due to its longer circulating half-life [19]. We therefore reexamined the distribution of the MacBlue transgene and relevant subset markers in the blood of control and CSF1-Fc-treated MacBlue mice (Figure 6). The administration of CSF1-Fc to MacBlue mice increased the total number of ECFP^+^ cells in the peripheral blood from 5.64±1% to 34.8±3.8%. Although both Gr1^+^(Ly6C/G^+^) and Gr1^−^ subsets were increased, the former were increased disproportionately (Fig 6A). The large majority of the ECFP^+^ cells expressed the macrophage marker F4/80 (Fig 6B) and CSF1R (CD115) (Fig 6C). Note that the CSF1-Fc had only a marginal impact on the proportions of Gr1^+^ECFP^−^ cells. We also identified ECFP^−^ cells that were positive for F4/80 (Fig 6B) and a smaller subset expressing CD115 (Fig 6C, upper left quadrant). The CD115^+^ cells were also expanded around 4-fold in the blood of CSF1-Fc treated mice (Fig 6C).

**Figure 7:**
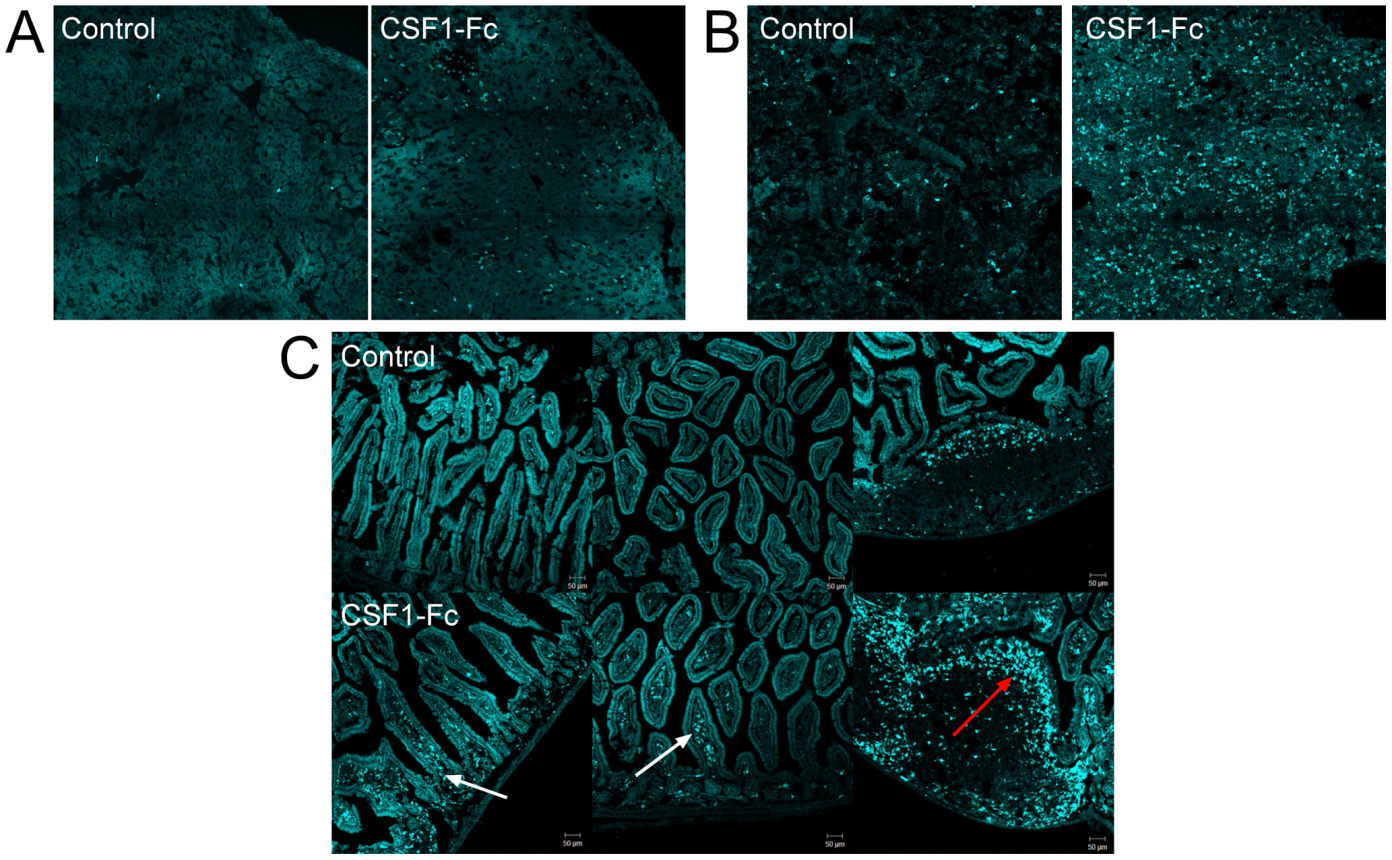
The expression of MacBlue transgenes in organs of mice injected with CSF1-Fc. Male and female MacBlue 8–15 weeks old were injected subcutaneously with either PBS or 1 mg/Kg porcine CSF1-Fc for 4 days prior to sacrifice on day 5. Perfusion fixed tissues were examined via confocal microscopy for ECFP expression. Representative images are shown. (A) liver, (B) lung and (C) lamina propria. White arrow: villi. Red arrow: sub-epithelial dome region of Peyer's Patch.

Consistent with the reported ability to promote monocyte infiltration, in the CSF1-Fc-treated mice, there was substantial infiltration of most tissues by ECFP^+^ cells. Figure 7 shows the liver (Fig 7A) and lung (Fig 7B). In the lamina propria of the gut, there was a massive infiltration of the sub-epithelial dome region of the Peyer's patch by ECFP^+^ cells in the CSF1-Fc-treated mice (Fig 7C). Increased numbers of positive cells were also seen within the villi.

### Tumour-associated macrophages

**Figure 8:**
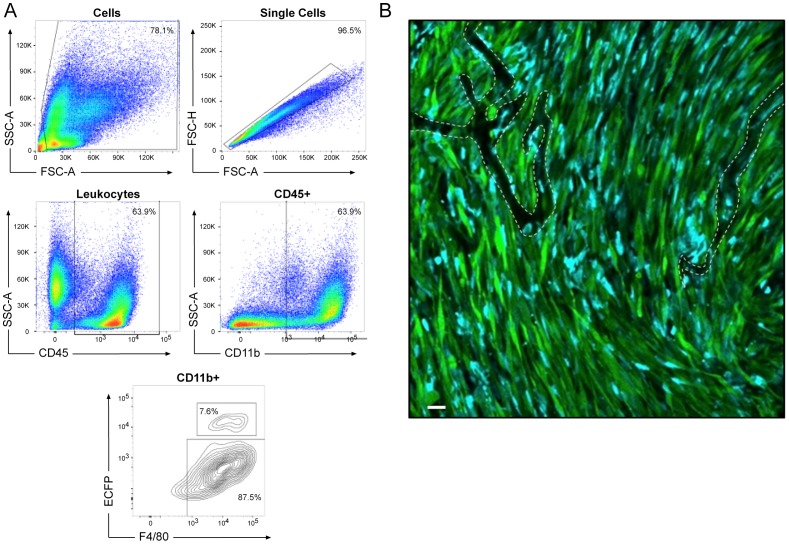
The expression of MacBlue transgenes in tumour associated macrophages. (A) Squamous cell carcinoma (SCC) tumours grown on the MacBlue mouse were removed, disaggregated and stained with CD45, CD11b, and F4/80 antibodies. FACS analysis of the resulting population using the gating shown was used to investigate the expression status of ECFP and F4/80 in the CD45^+^CD11b^+^ population. Results shown are representative of three tumours from three different animals. (B) Representative stitched confocal image of four adjacent images acquired through a dorsal skinfold window on an anesthetized mouse. Macrophages labelled with ECFP under the control of the Csf1r promoter were observed to infiltrate throughout the GFP-labelled SCC tumours. Blood vessels are outlined with a white dashed line. Scale bar 30 µm. Images representative of at least 5 separate tumours.

Our earlier studies demonstrated that anti-CSF1R treatment does not alter the recruitment of monocyte macrophages into inflammatory sites, but does greatly reduce the large numbers of tumour-associated macrophages (TAMs) detected using the MacGreen transgene [11]. We speculated that the MacBlue transgene would enable live imaging of monocyte infiltration in tumours. To enable the use of the MacBlue transgene in tumour biology, we bred it to the FVB background, where we have an established model of squamous cell carcinoma which attracts very large numbers of TAMs [37], [38]. Figure 8 shows representative FACS profiles of the purified TAMs, and an intravital image of their location within established tumours, revealing their close association with the vasculature. Analysis of surface markers of disaggregated leukocytes reveals that ECFP is expressed by only around 10% of the CD11b^+^, F4/80^+^ macrophages derived from the tumour.

### Summary

The MacBlue transgene is expressed in the earliest macrophage lineage cells in the embryo, and in foetal liver. In adults, the ECFP expression is retained only in blood monocytes, many classical DC, and in a small subset of MPS cells that have been reported to be established from embryonic sources (microglia, Langerhans cells, lung macrophages).

## Discussion

The conserved upstream region of the mouse *Csf1r* promoter contains TSS that are utilised by placental trophoblasts [7]. These sites are not used in humans, or larger mammals, which initiate transcription in placenta from a distal promoter 20 kb upstream in the 3′UTR of the *PDGFR* locus [39]. So placental transcription is unlikely to be the reason for their conservation. When the upstream promoter element was deleted in *Csf1r*-EGFP reporter mice, expression was ablated in trophoblasts, but also in OCL [23]. In the current study, we have recognised that the deletion also abolished expression in mature macrophages in many organs. This pattern clearly distinguished tissue macrophages in the adult from those in the developing embryo, where the transgene was first detected in the yolk sac and Reichert's membrane macrophages, and then expressed in foetal liver and in tissue macrophages until mid-development (Fig 1). Some recent data suggests that tissue macrophages in the adult can derive from yolk sac progenitors [8]–[9]. If this is indeed the case, the MacBlue transgene must be switched off in the tissue macrophages of the adult at some time late in gestation or after birth, since it is clearly strongly-expressed in embryonic tissue macrophages derived from both yolk sac and liver (Fig 1). Alternatively, it could provide a marker for a transition in the origin of tissue macrophages late in gestation. That possibility is the subject of ongoing investigation. The macrophages in the adult all express *Csf1r* mRNA, and are labelled with the intact *Csf1r* transgene in the MacGreen mouse. One feature that the MacBlue ECFP^+^ cells share is that they are the macrophage population that is largely retained in the CSF1-deficient *op/op* mouse [40]. So, one may consider the MacBlue transgene a novel marker for the CSF1-independent macrophage population.

Why should deletion of the distal promoter lead to a selective loss of expression in CSF1-dependent macrophages? Previous detailed *in vivo* foot printing analysis during macrophage differentiation did not extend to this region [41]. Analysis of human macrophages suggests there is significant protection from UV irradiation within the distal element [42]. Chromatin IP data reveals overlapping H3K4Me2 and H3K27 acetylation binding, indicate of active enhancers, extending through this region upstream of the macrophage TSS in thioglycollate-elicited murine macrophages [43]. The distal segment contains the elements required for growth factor-inducible expression of reporter genes in non-macrophage tumour cell lines, including signaling by ectopically-expressed CSF1R [44]. The most obvious explanation lies with the AP1 motif (TGACTCA in mice), which is conserved across mammals [23]. Nuclear AP1 activity is induced by CSF1 [45]. The AP1-binding transcription factor, c-jun, interacts specifically with PU.1 to promote macrophage differentiation and expression of other macrophage-specific genes ([46] and references therein) and jun kinase is required for CSF1-dependent macrophage survival [47]. Resident tissue macrophages express very high levels of *Csf1r* mRNA, and constantly internalize and degrade both ligand and receptor [12]. *Csf1r* mRNA transcription in BM-derived macrophages is repressed by CSF1, acting in part upon runx1 and the FIRE element within the first intron [48] which also contains a conserved and critical AP1 site [20]. Taking all of these data together, we speculate that the distal element is required to sustain *Csf1r* transcription in the presence of CSF1 in cells that depend upon CSF1 signaling for survival.

Langerhans cells and microglia are significantly reduced in the IL34 knockout mouse, in the latter case reflecting the region-specific expression of CSF1 and IL34 [49]. The view that microglia derive exclusively from yolk sac-derived progenitors early in development [50] has been widely-accepted although it contradicts much earlier studies of the origins of microglia in the brain [51] and retina [52] which demonstrated substantial infiltration of monocytes from the blood in the perinatal period in response to successive waves of cell death. In this respect, microglia may resemble Langerhans cells, the other macrophage population depleted in the IL34 null mouse [34]. The Langerhans cells of the skin appear early during embryonic development, but are mainly replaced by foetal liver-derived cells [53], [54]. Langerhans cells also retain the ECFP marker [24].

The largest F4/80^+^ macrophage population in the body is in the wall of the gut [55]. Classical DC from the intestine; F4/80^−^FcR^−^ cells defined by their ability to stimulate in an allogeneic mixed lymphocyte reaction, are a very minor subpopulation of the cells released by enzymatic disaggregation of the lamina propria [56]. There was a period of confusion about their identity derived from equating CD11c^+^ cells with DC [57]. CD11c is expressed on the large majority of myeloid cells in the lamina propria. Cells isolated by enzymatic digestion are a complex mixture, indeed the numbers of subsets that have been defined appears to be a function of the number of markers [58]. The ECFP^+^F4/80^−^MHCII/CD11c^hi^ “DC” that we observed amongst cells obtained by enzymatic disaggregation of the wall of the gut have been further subdivided based upon expression of CD11b and CD103 [59]–[61]. At least some of the cells in these populations expressed the MacGreen *Csf1r*-EGFP transgene and CSF1R [59], [60], although relatively few CD103^+^ cells expressed the EGFP reporter. The MacBlue ECFP marker enables visualization of the distribution of the classical DC. Bogunovic et al. concluded previously that the majority of CD103^+^, CD11b^−^ migratory “DC” derived from lymphoid tissue [59]. The distribution of the MacBlue transgene, combined with the FACS data, suggests that this is true for most of the classical DC in the wall of the gut, although the CD103^+^ cells might, like the macrophages from the lamina propria, down-regulate ECFP expression with time. The concentration of ECFP^+^ classical DC within isolated lymphoid follicles is consistent with immunohistochemical detection of CD11c^hi^, F4/80^−^ cells within these structures[62], [63]. The majority of *Zbtb46*-EGFP expressing cells in the gut wall are also found in Peyer's patches and isolated lymphoid follicles [33], [64]. The vast majority of the myeloid cells in the lamina propria, detected with the MacGreen EGFP transgene, but not the MacBlue ECFP, are also clearly F4/80^+^, MHCII^hi^ [56] and CSF1R-dependent [21] macrophages. They may have important localized functions in immunosuppression [65] and oral tolerance [66], [67].

The detailed analysis of peripheral blood in the MacBlue transgenic line (Fig 6) highlighted populations of cells that have commonly been overlooked in studies of monocyte biology. Mouse peripheral blood monocytes have been divided into two subsets based upon expression of the surface marker Ly6C, and the chemokine receptors CCR2 and CX3CR1 [68]. The populations also differ in the level of expression of F4/80 [21], [69]. Several lines of evidence indicate that these populations represent a differentiation series controlled by CSF1R signaling. Both of these populations express the MacGreen transgene in peripheral blood [21]. Also within MacGreen peripheral blood, there are EGFP^lo^ cells with substantially lower expression of F4/80 [21]. These cells were also evident in inflammatory exudates with foreign bodies or thioglycollate [21], [22] but expression was abolished when the 150bp upstream element is removed [23]. Isolation of EGFP^lo^ cells in inflammatory exudate revealed heterogeneous leukocytes with ring-shaped nuclei [22]. Others have noted that in terms of surface markers and morphology, such cells defy classification into granulocyte and monocyte subclasses [70]. They have some overlap with myeloid-derived suppressor cells [71] and a subset could include mobilized, Ly6C^+^, committed monocyte progenitors [72]. CSF1-Fc most likely promotes proliferation, differentiation, maturation and egress of cells from the BM at multiple stages of derivation from a pluripotent stem cell. In this respect, CSF1-Fc has effects in common with Progenipoietin (Flt3L/G-CSF) [69]. An earlier study of the response to a single dose of CSF1 in rats also concluded that the factor promotes release from the BM of monoblasts and pro-monocytes, and caused a monocytosis and neutrophilia [73]. CSF1 is also required for the transition from Ly6C^+^ to Ly6C^−^ monocytes [10]. Continued CSF1 signalling is not required for the production of blood monocytes but is clearly able to increase conventional monocyte numbers and can direct stem cell commitment [74].

The relative absence of detectable ECFP in most tissue macrophages of the MacBlue mouse, and also in the tumour-associated macrophages, where it is present on blood monocytes (including those within the tissues) could mean either that (a) the ECFP is rapidly extinguished as cells differentiate to become tissue macrophages or (b) the monocytic precursors of tissue macrophages are actually the F4/80^+^, CSF1R^+^ immature myeloid cells which lack ECFP (and which are also the precursors of mature monocytes) or (c) the tissue macrophages are maintained entirely by self-renewal. In the lamina propria of the intestine we do see evidence of heterogeneous expression of the MacBlue transgene (Fig 3), consistent with progressive loss from monocytes entering the tissue. In other tissues, macrophage populations may turn over more slowly, and/or down-modulate the EFCP more rapidly, so the lack of expression of ECFP cannot distinguish the models above. Tissue macrophages require continuous CSF1R signaling [21] and can proliferate in response to excess CSF1 [36]. Interpretations of recent data do not take account of the homeostatic role of CSF1. So, for example, the fact that monocyte depletion does not produce depletion of tissue macrophages [75] could simply mean that there is a mechanism of compensation via elevation of CSF1. What is clearly the case is that the MacBlue transgene provides a unique marker for the study of macrophage and DC biology including live imaging of processes of monocyte extravasation and migration and the response to CSF1 administration.

## Materials and Methods

### Mice

The *Csf1r*-Gal4VP16/UAS-ECFP^+^ (MacBlue) and *Csf1r*-EGFP^+^ (MacGreen) mice were bred, genotyped and obtained from The Roslin Institute Biological Research Facility. MacBlue mice were also bred onto a pure FVB background at the University of Edinburgh Biological Research Facility. Approval was obtained from The Roslin Institute and The University of Edinburgh Animal Welfare and Ethical Review Body. The experiments were carried out under the authority of a UK Home Office Project Licence under the regulations of the Animals (Scientific Procedures) Act 1986. Endotoxin-free porcine CSF1-Fc fusion protein was produced as described in detail elsewhere [19]. Male and female MacBlue mice (n = 16); 8–15 weeks old were injected subcutaneously with either PBS or 1 mg/Kg porcine CSF1-Fc for 4 days prior to sacrifice on day 5. Blood was collected by cardiac puncture or mice were perfused fixed with 4% paraformaldehyde.

### Embryo dissection and imaging

Embryos from timed matings were dissected using standard techniques as described in [76] and imaged using a StereoLumar v12 (Zeiss) microscope with ECFP and UV filters.

### Intestine imaging

Post euthanasia, ileal intestinal sections were excised, cut longitudinally and washed vigorously with ice cold PBS to remove all faecal matter and mucus. Sections were then whole-mounted onto slides with DAKO fluorescent mounting medium and tiled images of the en face view were visualised using a LSM710 (Zeiss) confocal microscope.

### FACS analysis of small intestine and colon

Lamina propria cells were obtained from adult mouse intestines by enzymatic digestion as described previously [28], [61]. Cells were isolated from mesenteric lymph nodes by enzymatic digestion with 1 mg/ml collagenase D (Roche) in calcium magnesium free (CMF) Hank's balanced salt solution (HBSS; Gibco, Invitrogen) for 45 minutes. After isolation, cells were passed through a 100 µm and a 40 µm filter before use (Corning).

For flow cytometric analysis of cells, 2–3×10^6^ cells were added to polystyrene tubes (BD Falcon), washed in ice cold FACS buffer (1 mM EDTA/2% FCS/PBS) and then incubated for 15 mins with purified anti-CD16/CD32 (Biolegend) to reduce non-specific binding via Fc receptors, then incubated with the following anti-mouse antibodies: rat CD45 (30-F11), hamster CD11c (N418), rat CD11b (M1/70), hamster CD103 (2E7; all Biolegend) and rat F4/80 (BM8), rat MHCII (M5/114.15.2) and rat Ly6C (HK1.4; all eBioscience). Cells were then washed in ice cold FACS buffer before analysis on a LSRII flow cytometer (BD Biosciences). Dead cells were excluded by including 7-aminoactinomycin D (Biolegend) in all staining panels. All data generated was analysed using FlowJo software (Tree Star Inc, OR, USA).

### Liver FACS analysis

Livers from Schedule 1 euthanized mice were digested in 2 mg/ml collagenase D (Sigma Aldrich) at 37°C for 30 min then passed through a 100 µm filter. Cells were centrifuged for 7 min at 50×*g* to remove hepatocytes. Further purification of nonparenchyaml cells was performed using a 30% percoll (Sigma Aldrich) gradient. Cells were stained with fixable viability dye eFluor 780 then incubated with Fc block (TrustainfcX, Biolegend) prior to staining with rat F4/80 (BM8, Biolegend), rat CD11b (M1/70.15, Invitrogen) and rat Ly6C (HK1.4, Biolegend). Flow cytometry performed using the LSR Fortessa and analysed using FloJo.

### Imaging of MacBlue mouse brains

For detection of microglia in the brain, 20 µm coronal brain sections were cut on a freezing sledge microtome and stored in cryoprotectant buffer (ethylene glycol and glycerol in phosphate buffer) at −20°C. For immunofluorescence staining, tissue sections were blocked with 5% normal donkey serum (Vector Labs, UK) in 0.3% triton/PBS for 1 hour. Sections were incubated with NeuN [1∶500] (Abcam) overnight at 4°C. The sections were washed before incubation with secondary antibodies conjugated with Alexa647 [1∶500] (LifeTech) for 2 hours at room temperature. Sections were mounted on to gelatin coated slides and coverslipped using ProLong Gold anti-fade reagent (LifeTech). Fluorescent images were taken using the LSM710 (Zeiss) confocal microscope and Zeiss Zen software.

### FACS analysis of MacBlue and MacGreen mouse brains

MacBlue and MacGreen mice were transcardially perfused with 0.9% saline and brain were removed and digested at 37°C for 1 h in an enzymatic cocktail consisting of 1x HBSS, 50 U/ml collagenase D, 100 µg/ml Nα-Tosyl-L-lysine chloromethyl ketone hydrochloride, 5 U/ml DNase I and 8.5 U/ml dispase. The tissue was then manually homogenised using a Dounce homogeniser. Myelin was removed after centrifugation of the homogenate on a 35% percoll (GE Healthcare) gradient. The resulting single cell suspension was incubated with 1 µg/ml anti-CD16/CD32 (Biolegend) before staining with a cocktail containing rat CD11b (M1/70 1∶500, Biolegend), rat CD45 (30-F11 1∶200, Biolegend) and rat F4/80 (BM8 1∶200, eBioscience). Flow cytometry was performed using a BD Fortessa and data was analysed using FlowJo V10 software.

### FACS analysis of blood from CSF1-Fc treated mice

100 µL of anti-coagulated (EDTA) whole blood was incubated with the appropriate antibody or isotype control in the dark at room temperature for 30 min: rat F4/80 Biolegend BM8 1∶200, rat Ly-6G/Ly-6C (Gr-1) Biolegend RB6-8C5 1∶200, rat CD115 (CSF1R) eBioscience AFS98 1∶1000, or Rat IgG2b, k Isotype Ctrl Biolegend RTK4530 1∶200). Blood was processed using DAKO Uti-lyse, erythrocyte lysing reagent (S3325) according to manufacturer's protocol. Samples were analyzed on BD LSR Fortessa (blood) and analysed using Summit 4.1 software (DAKO).

### Imaging tissues from MacBlue mice treated with CSF1-Fc

Tissues from perfused fixed MacBlue mice were placed into 4% PFA for 2 hours followed by overnight incubation in 18% sucrose at 4°C. The following day, tissues were embedded in Tissue-Tek OCT compound and snap frozen in isopentane. Frozen sections were cut (6-10 µm thick) at -16°C using a LEICA cryostat and mounted with DAKO fluorescent mounting medium (DAKO). The fluorescence of the ECFP was visualised using a Zeiss LSM710 confocal.

### FACS analysis of disaggregated SCC tumours

Tumours were established following injection of 1×10^6^ squamous cell carcinoma (SCC) cells into the flank of FVB MacBlue mice. Tumours were removed from euthanized mice and digested in DMEM supplemented with 2 mg/ml collagenase D for 1 hour at 37°C. Following centrifugation, cells were resuspended in blood cell lysis buffer (Pharm Lysis Buffer, Becton Dickinson) for 5 minutes at 37°C. Cells were then resuspended in PBS and passed through a 70 µm cell strainer and washed three times in FACS buffer (PBS+1% FBS+0.1% sodium azide). Cells were placed (1×10^5^ per well) in 96-well plates and cell pellets were resuspended in 50 µl of Fc block (Fc antibody (eBioscience) and incubated for 15 minutes at 4°C. 50 µl of antibody mixture (CD45 e780, aCD11b PE, and F4/80, all 1∶200) was added and samples incubated for 30 mins in the dark at 4°C. The plate was then centrifuged and the cells resuspended in FACS buffer for a total of 3 washes before analysis using a BD FACS Aria II. Data analysis was performed using FlowJo software.

### Intravital imaging

To enable intra-vital imaging of macrophage infiltration into tumours, a Green Fluorescent Protein (GFP) labelled tumour fragment was implanted as previously described under a dorsal skinfold window [38] on a MacBlue mouse and images acquired 9 days post-implantation. Briefly, tumours were first established by subcutaneous injection of GFP-expressing SCC cells [37] into the flank of an FVB mouse. After approximately 7 days the resulting tumour was removed from the euthanized host and small fragments of approximately 1 mm were implanted under a dorsal skinfold chamber that had been surgically installed onto an FVB MacBlue mouse using a surgical procedure previously described [38]. To enable high-resolution confocal imaging the mouse was anesthetized by isoflurane inhalation, placed on a custom built heated microscope stage, and the dorsal skinfold chamber mechanically clamped onto the stage. Images were acquired using an Olympus FV1000 confocal microscope equipped with a LUMPLFLN 40XW 0.8 N.A. water immersion objective lens. ECFP was imaged using a 405 nm laser line and GFP using a 488 nm laser line. Images were acquired using sequential scanning to avoid spectral bleed through from ECFP into the GFP detection channel.

### Imaging nasal-associated lymphoid tissue

The nasal-associated lymphoid tissue (NALT) was removed for analysis by first dislocating and removing the lower jaw. After removing cheek muscles, cheek bones and back teeth, the remaining nose part contained the nasal turbinates, septum, lateral walls, and palate. NALT was separated from the rest of the nasal tissue by peeling away gently from the palate and wholemounted onto microscope slides with DAKO fluorescent mounting medium. The oesophagus was carefully excised from the throat and cut open laterally. After gently washing with PBS, the oesophagus was wholemounted onto microscope slides with DAKO fluorescent mounting medium. All tissues were imaged using LSM710 confocal microscope.

## Supporting Information

Figure S1 **The expression of MacBlue transgenes in nasal-associated lymphoid tissue.** The oesophagus was excised from MacBlue mice and ECFP expression examined in the submucosa (A) and muscularis (B). The nasal-associated lymphoid tissue was also examined (C).
(TIF)

Figure S2 **The expression of MacBlue transgenes in dendritic cell populations of the gut.** Small intestinal, colonic and mesenteric lymph node isolates from MacBlue mice were obtained by enzymatic digest and analysed using multi-parameter flow cytometry. Live CD45^+^CD11c^+^MHCII^+^F4/80^−^ cells from small intestine (A) and colon (B) were split into 4 regions based on CD103 and CD11b expression and ECFP expression examined. (C) Cells were isolated from mesenteric lymph nodes by enzymatic digestion and resident DC (MHC^int/+^) and migratory DC (MHC^hi^) were examined for ECFP expression.
(TIF)
