# Supplementary figures and images for: The MacBlue Binary Transgene (csf1r-gal4VP16/UAS-ECFP) Provides a Novel Marker for Visualisation of Subsets of Monocytes, Macrophages and Dendritic Cells and Responsiveness to CSF1 Administration
Source: PLoS One. 2014 Aug 19;9(8):e105429. doi: 10.1371/journal.pone.0105429 (PMC4138162; doi:10.1371/journal.pone.0105429)

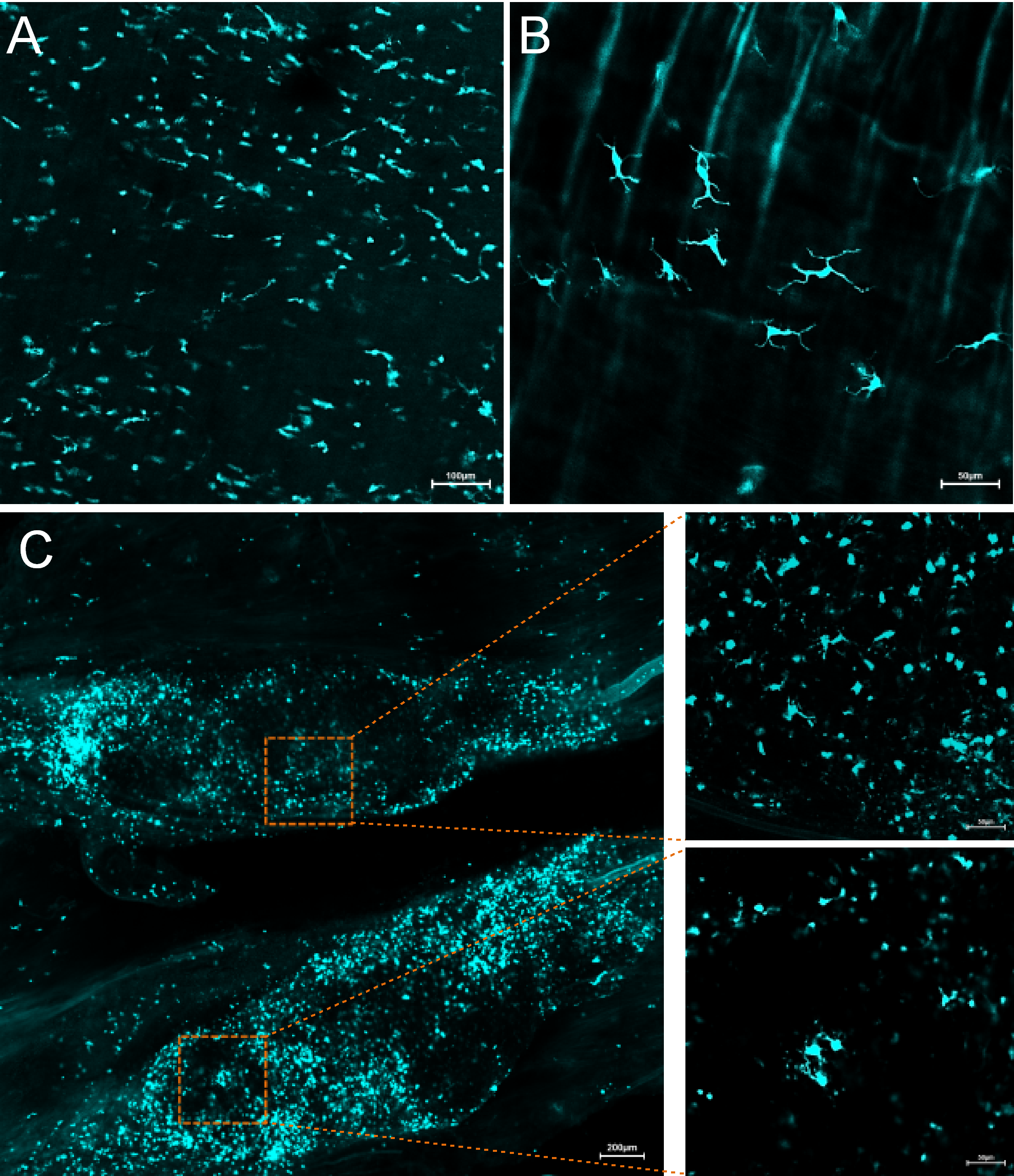

Supplement: Figure S1 — The expression of MacBlue transgenes in nasal-associated lymphoid tissue. The oesophagus was excised from MacBlue mice and ECFP expression examined in the submucosa (A) and muscularis (B). The nasal-associated lymphoid tissue was also examined (C). (TIF) [file pone.0105429.s001.tif]

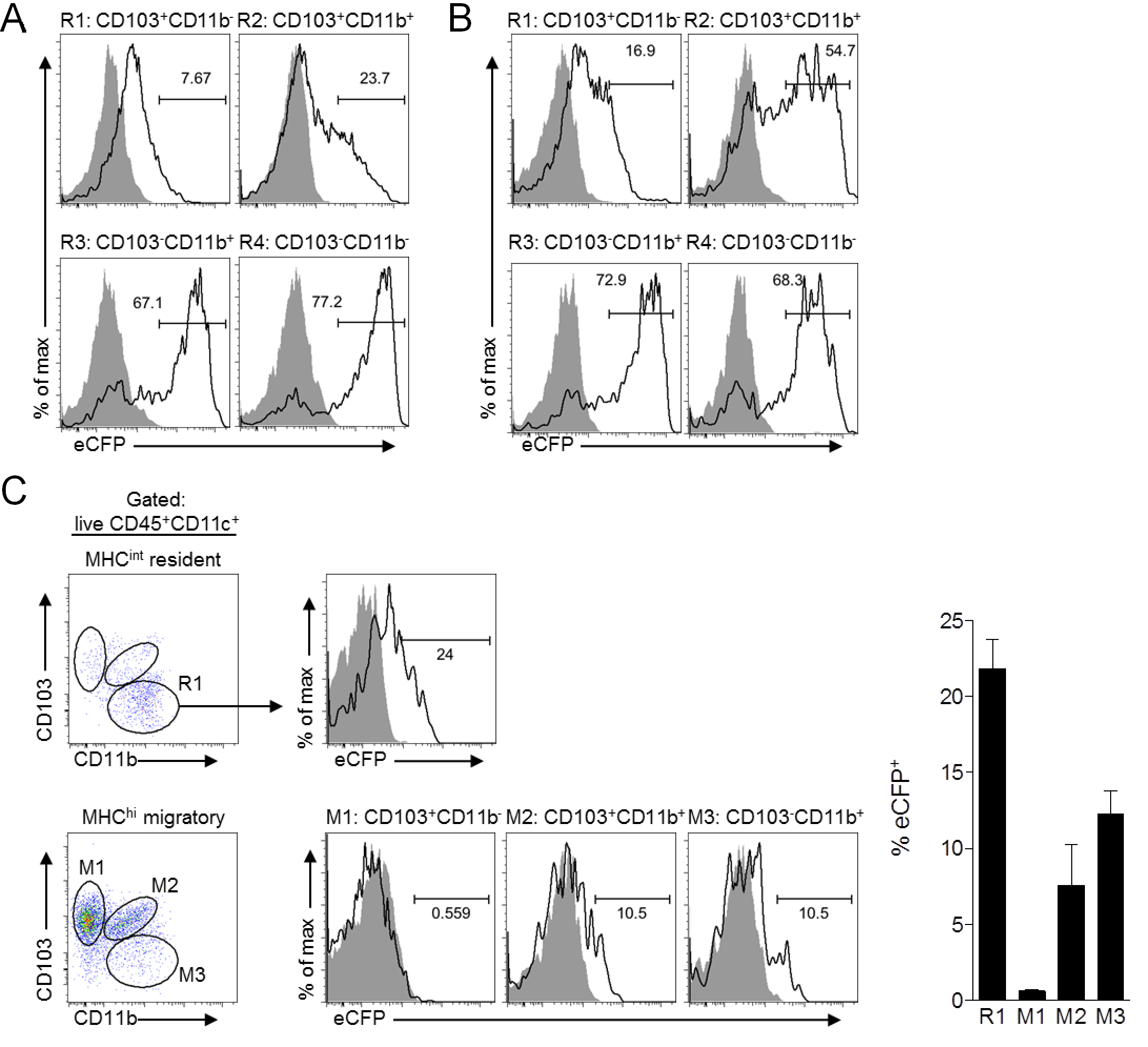

Supplement: Figure S2 — The expression of MacBlue transgenes in dendritic cell populations of the gut. Small intestinal, colonic and mesenteric lymph node isolates from MacBlue mice were obtained by enzymatic digest and analysed using multi-parameter flow cytometry. Live CD45+CD11c+MHCII+F4/80− cells from small intestine (A) and colon (B) were split into 4 regions based on CD103 and CD11b expression and ECFP expression examined. (C) Cells were isolated from mesenteric lymph nodes by enzymatic digestion and resident DC (MHCint/+) and migratory DC (MHChi) were examined for ECFP expression. (TIF) [file pone.0105429.s002.tif]
